# A critique of the WHO TobReg's "Advisory Note" report entitled: "*Waterpipe tobacco smoking: health effects, research needs and recommended actions by regulators*"

**DOI:** 10.1186/1477-5751-5-17

**Published:** 2006-11-17

**Authors:** Kamal Chaouachi

**Affiliations:** 1 Researcher in Socio-Anthropology and Tobaccology, Consultant in Tobacco Control, 62, avenue Victor Hugo; 92100 Boulogne Billancourt, France

## Abstract

**Background and aim:**

The World Health Organisation Study Group on Tobacco Product Regulation (TobReg) has issued in 2005 an "Advisory Note" entitled: "*Waterpipe Tobacco Smoking: Health Effects, Research Needs and Recommended Actions by Regulators*". "Waterpipe" smoking is now considered a global public health threat and the corresponding artefact is actually known in the world under three main terms: hookah, narghile and shisha. This important report, the first ever prepared by WHO on the subject, poses two major problems. On one hand, its bibliographical references dismiss world chief relevant studies. On the other, it contains a certain number of errors of many orders: biomedical, sociological, anthropological and historical. The purpose of the present study is to highlight, one by one, where these weaknesses and errors lie and show how this official report can be considerably improved.

**Results:**

We realise that widely advertised early anthropological studies were not taken into consideration whereas they shed a substantial light on this peculiar form of smoking and help understanding its high complexity. As for concrete errors to be found in this report, they deal with the chemistry of smoke, health-related effects, smoking patterns, description and history of the artefact and its use, gender and underage use aspects, prevention and research needs in this field.

**Conclusion:**

The scientific credibility of an international expert report may be at stake if its recommendations do not rely on sound objective research findings and a comprehensive review of the existing literature. The critical comments in this study will certainly help improve the present WHO report.

## Background

**Figure 1:**
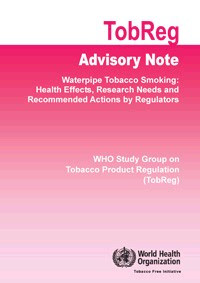
Cover of the WHO "Advisory Note" entitled "*Waterpipe Tobacco Smoking: Health Effects, Research Needs and Recommended Actions by Regulators*".

The World Health Organisation Study Group on Tobacco Product Regulation (TobReg) has issued in 2005 an "Advisory Note" entitled: "*Waterpipe Tobacco Smoking: Health Effects, Research Needs and Recommended Actions by Regulators*" [1](see Figure 1). This report was prepared "*in response to requests made by those Member States whose populations are exposed to this form of tobacco use*" and was adopted at a meeting in Rio de Janeiro on 7–9 June 2005. This document is mainly based on a background paper drafted by Dr Thomas Eissenberg (USA) and Dr Shihadeh (Lebanon), actually commissioned for this purpose by Dr Yumiko Mochizuki, Director of the WHO *Tobacco Free Initiative*, with the collaboration of Dr Maziak (Syria), Dr Israel and Dr Loffredo (USA) and Dr Mohamed (Egypt)[1]. Research led at the SCTS (Syrian Center for Tobacco Studies) formed an essential part of the report.

Such an official publication, which has remained uncommented so far, actually poses two major problems. The first one relates to its bibliographical references which dismiss world chief relevant studies, both in the biomedical and social sciences field. The case of the existing anthropological studies is particularly striking given the recognised importance of the socio-cultural dimension of this peculiar form of smoking. Indeed, the narghile practice is deeply rooted in a complex network of closely interrelated social, cultural and health issues in a given human context. The second problem deals with the multiple errors contained in the report. These are of many orders: biomedical, sociological, anthropological and historical.

Consequently, the aim of the present article is to highlight, one by one, where these weaknesses and errors lie and show how the WHO report can be considerably improved. We certainly share the health concerns stemming from the growing use of hookah (narghile, shisha, water-pipe) in the world. However, the scientific credibility of an international expert report may be at stake if recommendations do not rely on sound objective research findings.

In this study, we recall the importance and relevancy of the missing and glossed over references and critically review some of those cited by the authors. As for the errors, they are analysed under relevant subsections in the Results and Discussion section below.

## Results and discussion

### Origins

**Figure 2:**
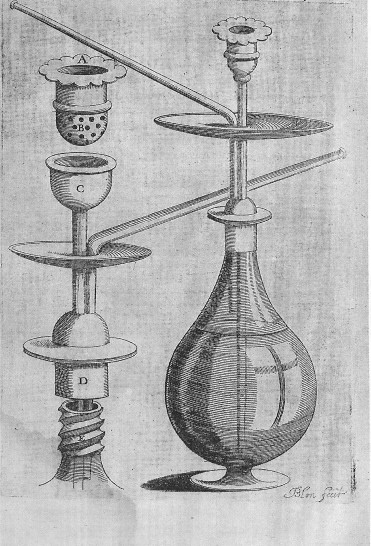
A Persian qalyân (1622). In *Tabacologia*, Johann Neander's masterpiece (1622). Drawing by Blon.

It is said (page 1) that, according to Chattopadhyay, "*waterpipes have been used to smoke tobacco and other substances by the indigenous peoples of Africa and Asia for at least four centuries*" [2]. The quotation is not accurate because this author does not mention, at any point, any of these facts in his article. Besides, evidence concerning the Indian origin of the hookah is weak. As a matter of fact, the most ancient traces were found in Southern or Eastern Africa [3]. For instance, bowls of water-pipes were dug out in 1971 by J.C. Dombrowski in the Lalibela cave (Ethiopia). C14 datation situated their use around years 1320 +/- 80 [3,4]. As for the large scale emergence of the narghile in society, either for an individual or collective use, historical accounts show that it was simultaneous with the appearance of the public coffee-houses and the adoption of tobacco in the Middle East region: near the end of the 16^th ^century and the beginning of the 17^th ^century (*ibid*.)(see Figure 2). For the authors of the report, the myth of the hookah as a safer way of smoking is as old as its invention in India. However, there is no point in insisting on the necessity of this Indian origin because it is also a myth itself.

### Tar yields

According to the report (page 1), "*Marketing tools associated with waterpipes and waterpipe tobacco may reinforce this unsubstantiated belief. For example, the label of a popular waterpipe tobacco brand sold in South-West Asia and North America states '0.5 nicotine and 0% tar*". An investigation was actually carried on with a similar product in France [3,5,6] and we had the possibility to clarify this point by stressing the importance of parameters such as the puffing frequency, volume and others (*id*.). These above figures were actually obtained with light smoking parameters similar to those used for cigarette smoking. Consequently, they do not reflect a less realistic view of human hookah smoking than others where the severe conditions imposed to a hookah smoking machine allowed the production, among others, of huge quantities of tar [7,8].

The quantity of tar obviously depends on the inhalation frequency. For instance two important, not cited, studies dealt with tumbâk [9,10] and used different puffing parameters than the recent one that relied on tobamel (tobacco-molasses smoking mixture, called "mu'essel" in Arabic)[8]. The discrepancies in results are striking: the two former found a reduction by c. 50% of the tar (smoke compared with that produced by a narghile with no water inside) while the latter succeeded in producing impressive amounts of it: 802 mg for a one-hour session. The explanation lies in the fact that, in the last case, the laboratory model completely differed from actual human hookah smoking. Only a small quantity of the smoking mixture was used: 10 g instead of the average 20 g according to an important work by Hadidi [11]. The coal, of the quick-lighting type, of unknown chemical composition, was kept in the same place over the tobacco-molasses mixture during the whole session, that is, almost one hour, versus 45 min [11]. This is contrary to the normal practice that involves moving it around in order to avoid "charring" the product. Puffs of a great volume (530 ml) were drawn periodically and at a fast pace: every 17 s vs. 30s [11]. In these conditions, 171 puffs were taken, vs. 90 [11]. These parameters were actually set according to a calculated average of figures collected through a smoking topography in a café. Strangely enough, the corresponding data were analysed, in their great majority, for the first 30 minutes of smoking only. Moreover, it is known that beyond that period, the interval between puffs tends to be much longer though remaining irregular. Over a whole session, the smoking variables have, randomly and in a drastic way, many ups and downs. Consequently, from a methodological standpoint, the use of an average figure over such a long span of time, particularly for puff frequency, is a serious methodological distortion. Indeed, specialists advise against the use of smoking machines in the field of cigarette, where, however, the smoking session is extremely short in comparison with the hookah: "*Kozlowski likened the FTC test to trying to measure caloric intake by inventing an eating machine. A better method he said, might be to study changes in people after eating. Similarly, studying the effects of cigarettes in actual people may be better than using a machine-based system. "In other words, cut out the machine smoker as the middleman," he said*" [12].

Moreover, there are also other important parameters that could change the amount and nature of the substances absorbed by the smoker: aspiration speed, pressure, water solubility of certain substances, volume of bowl, amount and temperature of water, added substances, length of the aspiration hose and others... What renders the tar dangerous is not its quantity but its quality and above all its temperature. In these conditions, the scientific truth is certainly to be found between these two extreme figures: neither "0% tar" as claimed on commercial tobamel packages nor "802 mg" artificially produced in unrealistic conditions in a laboratory.

As for nicotine, despite the above mentioned strain parameters, the nicotine yield, compared to that found in a single cigarette, is far from scaring the "hookah addicts" or even suggesting an explanation to their behaviour. Consequently, this issue shows that what is now needed is simulating, in a laboratory, the reality of human hookah smoking. If we want to be credible in our prevention efforts, artificial smoking, which generates great quantities of tar [7,8], should be avoided if we want to avoid confusion.

### Heating and burning

The report states (page 2) that "*the tobacco that is placed into the head is very moist (and often sweetened and flavoured): it does not burn in a self-sustaining manner*". This sentence is confusing. First, one should talk of diverse smoking concoctions or mixtures. Only one type of tobacco, tumbâk (which is raw tobacco), is "very" moist(ened) because, before it is packed inside the bowl, it is soaked in water then squeezed to remove most of its water. The other one, referred to as "*sweetened and flavoured*", is differently processed. It contains glycerol. Besides, in the case of the "flavoured tobacco", it is definitely wrong to say that the mixture is burnt. It is simply heated and this is a crucial point [13]. Evidence for this is provided by the actual working temperatures that can be measured during the process. They are below or around 100°C, a figure very different from that that can be measured at the tip of a cigarette (850–900°C). In these conditions, the heat range allows chemical reactions of the Maillard type between the aldehyde functions of sugars – especially in the molasses element- and nitrogenous compounds, particularly ammonia (NH4OH) used by tobacco manufacturers to produce various aromatic compounds [3,14]. Moreover, as far as tar is concerned, all specialists know that what makes the latter hazardous and carcinogenous is not its quantities but it qualities. On one hand, these qualities depend to a great extent on the temperature at which tar is produced. On the other, knowing the crude amount of tar is useless, because its harmful components, i.e. nitrosamines, polycyclic aromatic hydrocarbons, represent a very variable and, in any case, small percentage of the total weight, which therefore is in no way a valuable index of its hazards. This is true for cigarette smoke, so that one should stop printing on cigarette packs the smoking machine tar yield, which gives the smoker a fallacious information about the real danger. This is even more true for narghile smoking, which is much more variable. Once again, we can see that hookah smoking is very peculiar in this respect. In these conditions, even second-hand smoke (pages 4 and 5 of the report) is completely different from that produced by cigarettes [15].

### Children

We are informed (page 4) that "*in South-West Asia and North Africa, it is not uncommon for children to smoke with their parents", citing the *author of a so-called "dispatch" paper where we can read that: "*It is socially acceptable for a father to offer his teenage children a puff of the nargile, in a way similar to a Frenchman offering a taste of wine to his sons and daughters*" [16]. Despite the fact that this remark is quite personal and anecdotal, this does not mean at all that children "smoke with their parents", which is a totally unsupported statement in contradiction with all available anthropological data that rather describe narghile initiation as a kind of "rite of passage" [3,17].

More, recent epidemiological figures in Syria, an Arab country, are quite clear in this respect [18]: "*Age of initiation differed according to method of smoking and gender. On average, men initiated use of cigarettes at age 17.9 (5.3) years and waterpipe at 25.5 (9.1) years, while women initiated use of cigarettes at 22.5 (8.4) years and waterpipe at 28.9 (9.9) years (p, 0.05 for all gender and smoking method comparisons by t test*").

### Women

**Figure 3:**
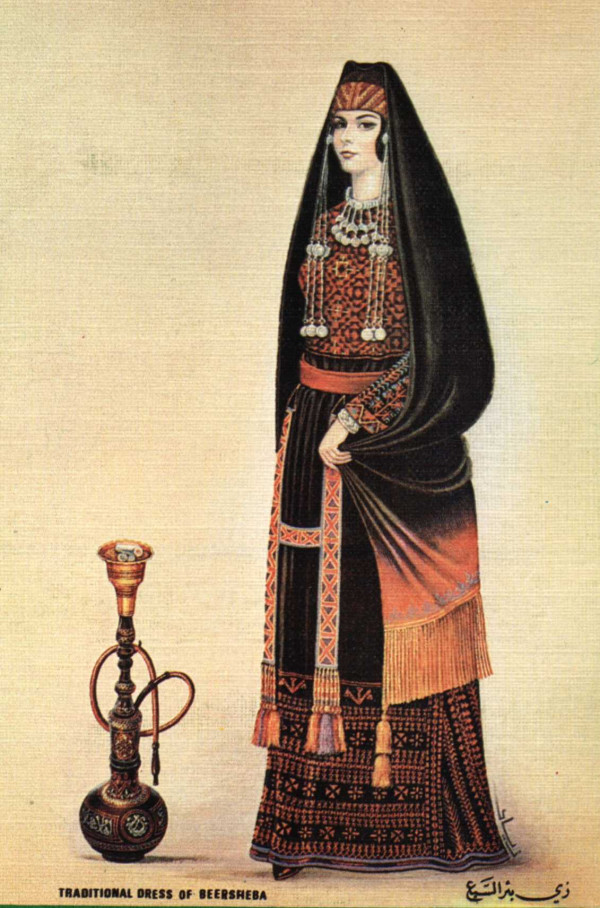
Traditional Palestinian Woman and Narghile.

In the same vein, we can read (page 4) that "*in some countries in which cigarette smoking is concentrated among men, waterpipe smoking appears more evenly distributed between both sexes*". This may be true in some countries but wrong in others. For instance, in Tunisia and Libya, cigarette smoking is still a male domain but "water-pipe" also. In these conditions, generalisation should be avoided because there is no "rule" in this field and this issue needs further comparativist anthropological research that already began for a decade now [15,19](see Figure 3).

### New charcoal

The report states (page 5, 5^th ^conclusion) that "*commonly used heat sources that are applied to burn the tobacco, such as wood cylinders or charcoal, are likely to increase the health risks because when such fuels are combusted they produce their own toxicants, including high levels of carbon monoxide, metals and cancer-causing chemicals*". These potential inherent listed hazards linked to the use of the new quick-lighting commercial charcoal are wrongly attributed to the two cited references [7,8]. As far as carbon monoxide is concerned, the above mentioned studies relied only on this new charcoal and neither a comparison with natural charcoal nor an analysis of the composition of the former was carried on. There is only one study in the world which shows that a commercial charcoal yields higher levels of this gas than the natural one [20].

Concerning heavy metals, the new quick-lighting charcoal might be a source but also the tobacco itself, the aluminium foil an even the metal coating (particularly of the bowl). Therefore, as there is no study to date about the composition and health effects of this new heating source, researchers are highly encouraged to set about clarifying this issue [3,5,6,13,21].

In any case, and if we only take two striking figures, that of the scaring yields for lead and carbon monoxide, 6870 ng and 143 mg respectively [7,8], they do not reflect at all the reality of hookah smoking. For instance, the results for the lead levels are not in agreement with a previous study by Salem concluding that the content of this metal was significantly higher in cigarette than in water pipe [22]. Other data from unpublished studies show that arsenic was not even detected. This is because the hookah smoking machine that was used [7,8] was set with blown up "average" parameters supposed to reflect figures varying, in fact, in a dramatic way and over a very long period of time (one hour). This is a distorted model of actual human hookah smoking behaviour [12].

### Harm reduction

In this field (page 5, 8^th ^conclusion), there would be "*no proof that any device or accessory can make waterpipe smoking safer*". For many reasons, there is no proof. For instance, and contrary to a widespread idea among tobacco control activists, the hookah market is still in the hands of "traditional" segments of the economy, particularly located in the so-called South. The producers of these devices do not possess the research facilities of an international company like RJ Reynolds, whose scientists publish their studies in international biomedical journals. For instance, there is a certain amount of literature about the Eclipse cigarette, based on the principle of narghile smoking because it heats tobacco instead of burning it. However, harm reduction techniques have already been put forward by the authors of high quality studies in the field of research on hookah smoking. For instance, Shafagoj in Jordan suggests to "*motivatemanufacturers to produce HB [hubble-bubble, i.e. hookah] filters to be inserted to the mouthpiece. In addition, pH, resins or other modifications can be made to the HB water to improve its filter properties*." [23]. In Arabia, Zahran early mentioned the existence of a device that obviously reduces, not to say inhibits, the production of carbon monoxide: an electric resistance used instead of the charcoal [24].

### Infections

The report states (page 5, 9^th ^conclusion) that "*sharing a waterpipe mouthpiece poses a serious risk of transmission of communicable diseases, including tuberculosis and hepatitis*". This statement is wrongly attributed to researchers [25] who are not the authors of studies on such risks. In the case of hepatitis, these studies were carried on by Habib et al. [26] and Medhat et al. [27]. As for tuberculosis, they were carried on, recently, by Munckhof et al. [28] and long before by Salem et al. [29]. Besides, the risk is not so "serious". If it had been so, the world would have witnessed corresponding epidemics over the last centuries. This did not happen. Finally, we note that plastic aseptic disposable personal nozzles are always served to patrons in the "hookah lounges" or during the fashionable home "hookah parties". Generally speaking, the risks are not clearly established because of a non-rigorous methodology (simultaneous use of other products [e.g. qât, cigarettes, bidis, etc], strongly neglected hygiene, current profile and remote and recent career of smokers not specified, etc.) [13,21].

### Scenario for the epidemic

We read (paragraph 10, page 5) that "*waterpipe tobacco is often sweetened and flavoured, making it very appealing; the sweet smell and taste of the smoke may explain why some people, particularly young people who otherwise would not use tobacco, begin to use waterpipes*". This quotation refers to a study that actually proposed a wrong "scenario" for the recent development of shisha use in Syria [30]. According to it, the Arab information satellite television channels would be greatly responsible for the development of the hookah craze. This argument is not consistent at all and the complex answer was treated in a key document [3]. The main reason is that the old Egyptian movies, that all television watchers of the Arab world remember quite well, were already heavily featuring narghile smokers long before the recent hookah epidemic and the emergence of satellite-powered TV channels. Moreover, it is also in contradiction with a paper cited as a key reference in the report and revealing: "*Old Egyptian films showed groups of men sipping mint tea or strong coffee in cafés and smoking nargile for many hours*" [16]. Unfortunately, this kind of quick analysis of a complex anthropological situation has led researchers of the very teams cited in this report to wade in a similar way [31].

### "Nicotine addiction"

We deem it not necessary to insist too much on the biochemical and health dimensions provided that the methodological conditions are respected in order to avoid the too frequent bias. Two critical reviews in French and Italian, among others, were published on these very aspects [3,5,6,13,21].

We also hope that in the coming future the nicotine addiction dogma will be sidestepped when tackling the dependence aspects. On one hand, it is not appropriate in the field of hookah smoking and the results of a study carried on by one of the very teams mentioned in this report, are quite clear: about 3/4 of the interviewees declared narghile use was easy to quit [32]. On the other, as far as dependence is concerned, there is a serious debate over the central role of nicotine in the dependence process [33,34]. Indeed, we are convinced that the future findings of the growing research on narghile will help reconsider the tobacco dependence issue in general and cigarette dependence in particular. People do not necessarily smoke the hookah for nicotine and another evidence for this is that the "hookah lounges" already offer herbal fruit-flavoured tobacco-free smoking mixtures to their patrons. The importance of flavours (only in the case of tobamel) would make this dependence very similar to that induced by coffee. Not only nicotine but MonoAmine Oxidase Inhibitors (MAOI), other minor low-dose potentially dependence-inducing alkaloids, ligands of opioid receptors, and other substances, might play a certain role in the dependence process [34].

### Chief studies

Globally, we may wonder why controversial studies [7,8,35,36] are cited in this report while other early and useful studies led by Hoffman [9], Rakower [10] and Salem [22,29] are not. This is all the more regretful that the latter relied on traditional settings and the corresponding experiments were based on the use of the natural charcoal, not the new self-lighting one which likely causes, among others, an overproduction of carbon monoxide [20]. Indeed, what is interesting for the sake of scientific comparisons, is that one basic difference between the traditional four-century old social use of the hookah and the contemporaneous one is the nature of the heating source.

### Prevention

Prevention is the most important and pressing issue and no suggested action (page 7) is given in this report whereas a public health catastrophe is looming and so many ideas could immediately be put forward to avoid it and reduce the harm caused by this new widespread form of smoking [3,5,6,13,21,37].

### State of research

The report states (page 6) that "*there is surprisingly little research addressing tobacco smoking using a waterpipe, especially given that there are many millions of current waterpipe smokers and that waterpipe use is spreading across the globe*". It is true that very little research has been devoted to the subject. Therefore to perform an exhaustive bibliography was not a superhuman task, and the WHO report should have done it. We personally regret that the authors did not even mention the deep and early health oriented anthropological research that we carried on in this field, which contains many ideas for the desired development of prevention and cessation strategies" [3,5,6,13,21]. These documents have been widely advertised, over the years 2000–2005, among the international community of tobacco control researchers and activists, particularly through the Globalink network. English abstracts, translations and comments of their findings were disseminated on a large scale.

### Drugs

One can also read that there would be research needs for "*the relationship between waterpipe smoking and the use of other drugs, including marijuana*" (page 6). We will point out that a 262 page scientific book, entirely dedicated to this topic, was published as early as 1997 [38].

## Conclusion

We hope these comments will be useful for the future reports which, we insist, should be clear and objective, if we want them to be accepted, as far as their recommendations are concerned, by health practitioners, prevention activists, researchers and the one-day or regular smokers themselves all over the world. Methodology needs substantial improvement. The World Health Organisation Study Group on Tobacco Product Regulation itself, responsible for the publication of this very report actually concluded in a meeting: "*The International Organization for Standardization (ISO) machine-based cigarette test for determining the tar, nicotine, and carbon monoxide content of cigarettes should be banned and replaced*" [12]. What is true for a 5 minute cigarette smoking session is even much more true for hookah smoking session which is 10 times longer and where the puffing variables extremely deviate from an arbitrary "average" figure. In these conditions, the use of a hookah smoking machine [7,8] should be discontinued because it has led to a great deal of confusion, particularly reflected in the first report on "waterpipe smoking" ever published by WHO [1]. The 12^th ^core principle set out in another TobReg important report is: "*Regardless of the funding mechanism adopted, it should ensure that the independence and integrity of research and testing operations are not compromised or inappropriately influenced *» [39]. The same document insists that the output of research and testing laboratories be "credible and consistent with the most rigorous of international standards" (p.7, *ibid*.).

We invite our colleagues, particularly those working in the epidemiological field, to amend the design of their questionnaires and include one or several items related to the past and present career (ex-smoker [with quantification, detailed mention of dates an products], switching-smoker from cigarette to narghile, exclusive smoker, etc.) of the volunteers analysed in their studies [5,21,37]. This point is of utmost importance because it may render useless the corresponding results as this happened with most of the previous studies. In view of the quick development of hookah use in the world, we will definitely spare time and other resources for the benefit of the world human health. On one hand, the strong socio-anthropological background of fashionable hookah smoking in a global world shows that the underlying social context bears similarities but also great differences with cigarette smoking [41]. On the other, it confirms that "public health is a social issue" as recently and relevantly emphasised by Dr Lee, Director-general of the World Health Organisation [42].

## Methods

The present work is based on a critical and thorough review of the WHO report. The findings of the studies cited by the authors are compared with the results of those not cited in that document, both in the biomedical and social sciences fields. We realised that the errors concretely deal with the following themes: the chemistry of smoke, health-related effects, smoking patterns, description and history of the artefact and its use, gender and underage use aspects, prevention and research needs in this field. Their corresponding analysis and comments are combined as far as possible and reported as subsections in the Results and Discussion section, namely: Origins; Tar yields; Heating and burning; Children; Women; New charcoal; Harm reduction; Infections; Scenario for the Epidemic; "Nicotine addiction"; Chief studies; Prevention; State of research; Drugs.

## Competing interests

The author(s) declare that they have no competing interests.
